# Diffusion Microstructure Imaging to Analyze Perilesional T2 Signal Changes in Brain Metastases and Glioblastomas

**DOI:** 10.3390/cancers14051155

**Published:** 2022-02-23

**Authors:** Urs Würtemberger, Martin Diebold, Daniel Erny, Jonas A. Hosp, Oliver Schnell, Peter C. Reinacher, Alexander Rau, Elias Kellner, Marco Reisert, Horst Urbach, Theo Demerath

**Affiliations:** 1 Department of Neuroradiology, Medical Center—University of Freiburg, Faculty of Medicine, University of Freiburg, 79106 Freiburg, Germany; alexander.rau@uniklinik-freiburg.de (A.R.); horst.urbach@uniklinik-freiburg.de (H.U.); theo.demerath@uniklinik-freiburg.de (T.D.); 2 Institute of Neuropathology, Medical Center—University of Freiburg, Faculty of Medicine, University of Freiburg, 79106 Freiburg, Germany; martin.diebold@uniklinik-freiburg.de (M.D.); daniel.erny@uniklinik-freiburg.de (D.E.); 3 IMM-PACT Clinician Scientist Program, Faculty of Medicine, University of Freiburg, 79106 Freiburg, Germany; 4 Berta-Ottenstein-Program for Advanced Clinician Scientists, Faculty of Medicine, University of Freiburg, 79106 Freiburg, Germany; 5 Department of Neurology and Neurophysiology, Medical Center—University of Freiburg, Faculty of Medicine, University of Freiburg, 79106 Freiburg, Germany; jonas.hosp@uniklinik-freiburg.de; 6 Department of Neurosurgery, Medical Center—University of Freiburg, Faculty of Medicine, University of Freiburg, 79106 Freiburg, Germany; oliver.schnell@uniklinik-freiburg.de; 7 Department of Stereotactic and Functional Neurosurgery, Medical Center—University of Freiburg, Faculty of Medicine, University of Freiburg, 79106 Freiburg, Germany; peter.reinacher@uniklinik-freiburg.de (P.C.R.); marco.reisert@uniklinik-freiburg.de (M.R.); 8 Fraunhofer Institute for Laser Technology, 52074 Aachen, Germany; 9 Department of Diagnostic and Interventional Radiology, Medical Center—University of Freiburg, Faculty of Medicine, University of Freiburg, 79106 Freiburg, Germany; 10 Department of Medical Physics, Medical Center—University of Freiburg, Faculty of Medicine, University of Freiburg, 79106 Freiburg, Germany; elias.kellner@uniklinik-freiburg.de

**Keywords:** glioblastoma, cerebral metastasis, diffusion magnetic resonance imaging, diffusion tensor imaging

## Abstract

**Simple Summary:**

Glioblastomas and brain metastases are the most common malignant intracerebral tumors in adults and are often difficult to differentiate using conventional MRI. Common to both is contrast enhancement on T1w post-Gd sequences and perilesional T2 hyperintensity, which in glioblastomas corresponds to diffuse tumor infiltration rather than to edema alone. Characterization of perifocal T2 hyperintensity in glioblastoma has been poor using MRI, and tumor recurrences frequently occur in these areas. Using a novel diffusion microstructure imaging (DMI) approach, we observed that in metastases, perilesional T2 hyperintensity is more likely due to edema alone with a higher fraction of free water and reduced cellular compartments compared to glioblastomas. DMI might be a powerful diagnostic tool, as it could be used to distinguish metastases from GBM, as well as characterizing perifocal T2 hyperintensity in GBM.

**Abstract:**

Purpose: Glioblastomas (GBM) and brain metastases are often difficult to differentiate in conventional MRI. Diffusion microstructure imaging (DMI) is a novel MR technique that allows the approximation of the distribution of the intra-axonal compartment, the extra-axonal cellular, and the compartment of interstitial/free water within the white matter. We hypothesize that alterations in the T2 hyperintense areas surrounding contrast-enhancing tumor components may be used to differentiate GBM from metastases. Methods: DMI was performed in 19 patients with glioblastomas and 17 with metastatic lesions. DMI metrics were obtained from the T2 hyperintense areas surrounding contrast-enhancing tumor components. Resected brain tissue was assessed in six patients in each group for features of an edema pattern and tumor infiltration in the perilesional interstitium. Results: Within the perimetastatic T2 hyperintensities, we observed a significant increase in free water (*p* < 0.001) and a decrease in both the intra-axonal (*p* = 0.006) and extra-axonal compartments (*p* = 0.024) compared to GBM. Perilesional free water fraction was discriminative regarding the presence of GBM vs. metastasis with a ROC AUC of 0.824. Histologically, features of perilesional edema were present in all assessed metastases and absent or marginal in GBM. Conclusion: Perilesional T2 hyperintensities in brain metastases and GBM differ significantly in DMI-values. The increased free water fraction in brain metastases suits the histopathologically based hypothesis of perimetastatic vasogenic edema, whereas in glioblastomas there is additional tumor infiltration.

## 1. Introduction

Glioblastoma (GBM; IDH-wildtype (IDH-wt)) is the most common malignant primary intracerebral tumor in adults [1]. Despite intensive research efforts and current standard therapy with radical tumor resection and adjuvant radiochemotherapy, the prognosis is poor with a median survival of 14.6 months [2]. Tumor infiltration in GBM usually extends well beyond the contrast-enhancing and even T2 hyperintense tumor portions, and to date has been difficult to characterize by conventional MR imaging. The assumption that the contrast-enhancing margin of GBM does not correspond to its tumor boundary is supported by the fact that most tumor recurrences occur within peripheral T2/FLAIR hyperintense areas [3]. It is, therefore, necessary to further characterize infiltrative GBM tumor components by MRI.

Brain metastases are the most common intracranial malignant tumors in adulthood and predominantly arise from five primary tumors [4] (lung cancer, breast cancer, malignant melanoma, gastrointestinal tract tumors, and renal cell cancer) and may show marked heterogeneity on MR imaging. In particular, cerebral metastases with marginal contrast enhancement, perifocal T2/hyperintensity, and solitary localization often cannot be reliably distinguished from high-grade gliomas such as GBM by conventional MR imaging.

Since the treatment regimens and prognoses of GBM and metastases differ, a reliable non-invasive differentiation is of great relevance. In GBM, the goal is radical complete tumor excision of contrast-enhancing tumor portions, whereas in metastases, the focus is on systemic diagnosis and therapy. Therefore, in cases of uncertainty, stereotactic tumor biopsy is usually performed, which carries risks depending on factors such as tumor location and patient age and always involves the possibility of sampling error [5,6]. It would be helpful in clinical routine to be able to distinguish GBM and metastases more precisely with noninvasive techniques such as MRI.

Advanced diffusion imaging has undergone steady development within the past decades from diffusion tensor imaging (DTI) to novel techniques such as neurite orientation and dispersion imaging (NODDI) [7], and diffusion microstructure imaging (DMI) [8]. Numerous studies have used diffusion imaging techniques to try to distinguish between brain metastases and GBM/high-grade gliomas [9,10,11] under the assumption that diffusion parameters differ between predominantly vasogenic edema, such as those in brain metastases, and vasogenic edema with additional tumor infiltration, such as those in GBM [12,13]. However, due to divergent results, these parameters are not established in routine diagnostics yet [9,14,15]. For example, Wang and colleagues found no significant differences in fractional anisotropy (FA) in the peritumoral edema between GBM and brain metastases, and they concluded that no DTI–metric threshold was able to distinguish between peritumoral infiltrative edema and vasogenic edema [11]. Another recent study employed various diffusion techniques including NODDI, DTI, and classical DWI and was able to identify significant differences within the contrast-enhancing tumor regions, but no significant alterations within the perilesional T2 hyperintense area [16].

DMI is a novel technique, similar to NODDI. It allows the differentiation of three microstructural components based on their different diffusion properties. Here, the intra-axonal volume compartment can be distinguished and relatively quantified from the extra-axonal intracellular and extracellular free water compartments [7,8,17]. While water molecules in neuronal structures or the extracellular matrix are aligned by organelles and membranes, free water molecules are modelled to move randomly and unimpeded. From this, DMI derives a standard model for white matter consisting of the intra-axonal volume fraction (V-intra) with nearly one-dimensional molecular diffusion due to the narrow membrane boundaries, and the extra-axonal volume fraction (V-extra) corresponding to the extra-axonal cellular compartment and the extracellular matrix and characterized by restricted diffusion and the free water/CSF fraction (V-CSF). DMI has recently been used in clinical research to study normal pressure hydrocephalus (NPH) [18] and temporal lobe epilepsy [19] and is a promising diffusion imaging technique that could find application in numerous white matter pathologies.

Considering the different edema characteristics in GBM and metastases (infiltrative vs. vasogenic), the aim of this study was to analyze, whether the perilesional diffusion microstructure imaging metrics V-intra, V-extra, and V-CSF differ between GBM and metastases and to correlate the findings histopathologically.

## 2. Materials and Methods

### 2.1. Patient Selection

Patients presenting with an intra-axial contrast-enhancing lesion were retrospectively included within a one-year period (2020–2021). The prerequisite for inclusion was the presence of a contrast-enhancing intra-axial lesion, with perilesional T2 signal elevations in the white matter. Patients with severe small vessel disease (Fazekas > 1), concomitant vascular lesions (e.g., vascular malformations), or neurodegenerative disorders (e.g., Alzheimer’s disease, frontotemporal lobar degeneration (FTLD), cerebral amyloid angiopathy) were excluded. Further exclusion criteria were previous radiation therapy, resection, or biopsy and poor image quality due to motion artifacts.

Imaging was performed with a dedicated tumor protocol on a 3 Tesla scanner (MAGNETOM Prisma, Siemens Healthcare, Erlangen, Germany) using a 64-channel head and neck coil including a diffusion tensor/microstructure imaging (DTI/DMI) sequence and isotropic T2w FLAIR and T1w MPRAGE sequences for anatomical delineation and segmentation. Post-contrast T1w sequences were acquired 4–5 min after intravenous injection of 0.1 mmol/kg gadobutrol (ProHance^®^, Bracco Imaging, Milan, Italy). The sequence parameters are presented in Table S1.

The study was conducted in accordance with the 1964 Helsinki Declaration and its later amendments and approved by the local ethics committee. Informed written consent was waived by the local ethics committee (Ethics Committee-Freiburg University Medical Center) due to the purely retrospective analysis. We hereby confirm that all methods were performed in accordance with the relevant guidelines and regulations.

### 2.2. Diffusion Microstructure Imaging (DMI) and ROI Based Analysis

All data processing was implemented within our in-house post-processing platform NORA (www.nora-imaging.org; last accessed on 2 January 2022). Preprocessing of diffusion-weighted images included a denoising step [20] followed by correction of the Gibbs-ringing artifacts [21] and final upsampling to isotropic resolution [8].

**Figure 1:**
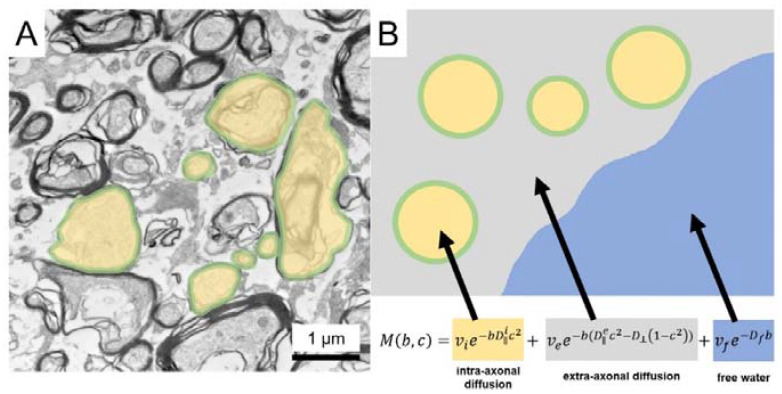
MR-physical concept of diffusion microstructure imaging. White-matter electron microscopic (60 nm ultrathin sections) (**A**) and schematic representation (**B**). Briefly summarized, the microstructure model can be traced back to a 3-compartment model in which *D* and *v* describe the diffusivities and the volume fractions of the corresponding compartments, where the subscript *i* refers to the intra-axonal compartment, *e* to the extra-axonal compartment, and *f* to the free water compartment. Visualization of the extra-axonal and free water compartments are anatomically compromised by preparation artifacts in electron microscopic sections (**A**). With permission by [19].

**Figure 2:**
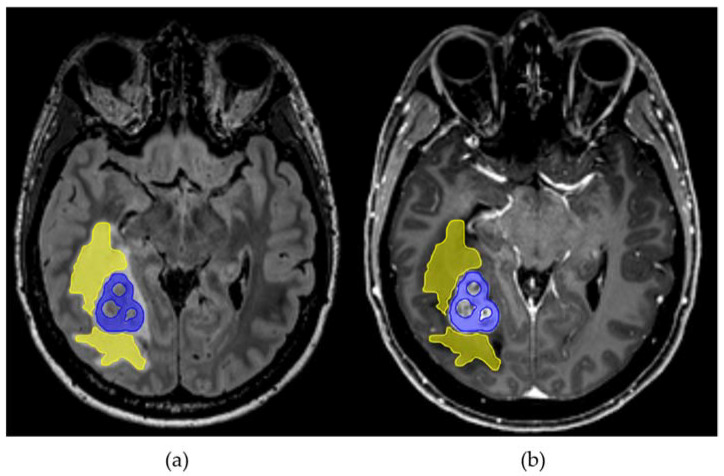
Axial FLAIR (**a**) and T1 MPRAGE post-Gd (**b**) images in a patient with a right occipital brain metastasis with corresponding perilesional FLAIR-hyperintensity (yellow) and contrast-enhancing tumor (blue) segmentations.

Microstructural diffusion metrics based on a three-compartment diffusion model were estimated using a Bayesian approach [8] to determine the intra-axonal volume fraction (V-intra), the extra-axonal intracellular volume fraction (V-extra), and the extracellular (CSF/free water = V-CSF) volume fraction (Figure 1). T1w imaging datasets were automatically segmented into white matter, grey matter, and cerebrospinal fluid (CSF) using SPM12 (Wellcome Centre for Human Neuroimaging, London, UK). Perilesional T2w white matter hyperintensities were manually segmented on 3D reformatted FLAIR images with co-registration with T1 post-Gd datasets to avoid accidental segmentation of contrast-enhancing tumor components (Figure 2). SPM12 segmentation maps of white matter with the exclusion of T2w-hyperintense areas were defined as whole-brain normal appearing white matter (NAWM). Diffusion metrics were normalized to the NAWM in each case, accounting for age-related white matter changes. To account for potential steroid-related effects, analyses were complemented by excluding patients with corticosteroids, since doses and temporal relation to time interval before imaging were not standardized.

### 2.3. Histopathology

Histological analysis of available biopsy tissue followed standardized protocols of the local institute of neuropathology. Sample acquisition followed established diagnostic procedures for fixation in 4% paraformaldehyde, paraffin embedding, and staining. Sections (4 μm thick) were processed and stained with hematoxylin and eosin (H&E). All available biopsy material containing adequate fractions of both tumor tissue and adjacent CNS parenchyma were considered for analysis (available in 6 GBM cases and 6 metastasis cases). Vasogenic edema was assessed on H&E sections by analysis of accentuated tissue disaggregation and increase in translucent areas [22]. Exemplary images were obtained using an Olympus BX40 microscope (Olympus, Shinjuku, Tokyo, Japan) and a Leica DFC450 camera (Leica, Wetzlar, Germany). The samples analyzed in this study had been obtained from surgeries that aimed for radical removal of contrast-enhancing tumor portions, so perilesional T2 changes were not targeted for resection. Therefore, usable histopathologic tissue samples could not be obtained for all tumors studied.

#### Statistical Analysis

Normal distribution assessed by the Shapiro–Wilk test. The Mann–Whitney-U test was employed to compare age and perilesional T2 volumes between GBM and metastasis groups. A One-way ANCOVA, controlling for lesion volume and Bonferroni correction for multiple comparisons was conducted between perilesional T2 areas comparing GBM and metastasis groups. The Pearson’s correlation coefficient was used to relate V-intra, V-extra, and V-CSF to T2 volumes. We plotted the receiver operating characteristic (ROC) curves of GBM and metastasis perilesional and V-extra, V-intra, and V-CSF. Values with an α-level of 0.05 were considered statistically significant. All statistical analyses were performed using R statistics V. 4.0 (R Core Team 2020, Bell Laboratories, Holmdel, NJ, USA; https://www.R-project.org; last accessed 18 February 2022). Boxplots were calculated using CRAN.R-packages (https://CRAN.R-project.org/package=ggplot2 last accessed 18 February 2022, https://CRAN.R-project.org/package=ggstatsplot last accessed 18 February 2022). The Test ROC module was used to calculate ROC curves, which is built on the cutpointR module version 1.1.1 (https://CRAN.R-project.org/package=cutpointr last accessed 18 February 2022).

## 3. Results

### 3.1. Study Population

Out of 52 patients presenting with contrast-enhancing intracranial mass lesions, 17 patients (8 female; mean age: 63.5; SD 11.8, range 47.1–85.6 years) with histologically verified brain metastases and 19 patients with GBM (IDH wildtype, 9 female; mean age: 66.4; SD 14.1, range 41.8–88.0 years) underwent presurgical MRI. The following characteristics led to exclusion from the analyses: previous brain surgery (*n* = 7) and/or radiotherapy (*n* = 3), IDHmut high-grade gliomas (*n* = 2; excluded according to the current classification WHO 2021), limited image quality which prevented image postprocessing (*n* = 4). Corticosteroids had been administered in 7 patients in the GBM group and 6 patients with metastases. Due to the retrospective evaluation, the exact temporal relation of steroid administration to the time of imaging could not be determined. Primary tumors in patients with brain metastases were lung cancer (*n* = 10), melanoma (*n* = 3), urothelial carcinoma (*n* = 1), colorectal carcinoma (*n* = 1), esophageal carcinoma (*n* = 1), ovarian cancer (*n* = 1). The groups did not differ concerning age (*p* = 0.44) or total volume of tumor-related T2 signaling changes (*p* = 0.93).

### 3.2. Diffusion Metrics in Brain Metastasis and GBM

**Figure 3:**
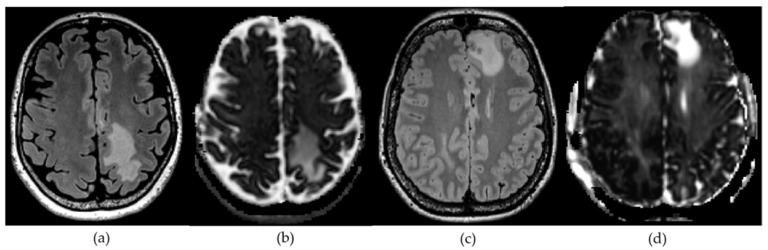
Axial FLAIR (**a**,**c**) and parametric V-CSF-maps (**b**,**d**) in a left centroparietal GBM (**a**,**b**) and left frontal metastasis (**c**,**d**). Of note, the metastasis shows a relative increase in perilesional V-CSF compared with GBM (**d**,**b**).

**Figure 4:**
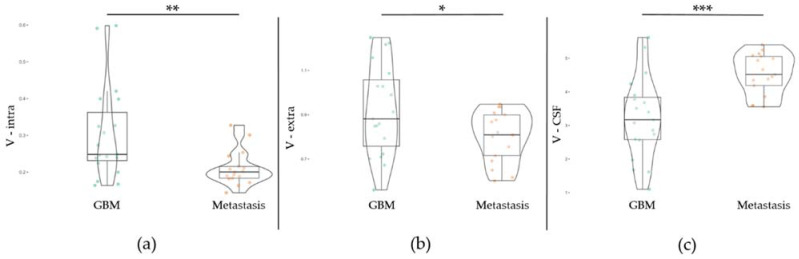
Normalized perilesional (T2) diffusion microstructure imaging (DMI) metrics in patients with GBM (*n* = 19) and metastases (*n* = 17), normalized to whole-brain normal-appearing white matter (NAWM) values. Compared to GBM, metastases show a significant shift towards increased interstitial free water ((**c**); V-CSF) and decreased V-intra and V-extra (**a**,**b**). * *p* ≤ 0.05; ** *p* ≤ 0.01; *** *p* ≤ 0.001.

The DMI parameters of the T2 hyperintense area were normalized by the values of whole-brain NAWM and compared between GBM and metastasis groups (exemplary in Figure 3), controlling for perilesional T2 hyperintense area volume. Within the perilesional T2 hyperintense area a significant increase in V-CSF in metastases compared to GBM, (F (1,1) = 17.11, *p* < 0.001), a decrease in V-extra (F (1,1) = 5.60, *p* = 0.024) and V-intra (F (1,1) = 8.71, *p* = 0.006) was found. Group-related metrics and ranges are presented in Table 1. The distribution of the individual values is shown in Figure 4. A further analysis excluding patients with corticosteroid treatment led to similar results with an increase in V-CSF in metastases compared to GBM, (F (1,1) = 8.48, *p* = 0.009) and a decrease in V-extra (F (1,1) = 6.91, *p* = 0.016) and V-intra (F (1,1) = 9.68, *p* = 0.005).

### 3.3. Correlation and ROC Analysis

Results of the Pearson’s correlation indicated a positive association between perilesional T2 volume and V-CSF in metastases (r = 0.51, *p* < 0.036), without reaching significance in GBM (r = 0.33, *p* = 0.170). Furthermore, in both groups we found a negative correlation between patient age and perilesional V-CSF (metastases: r = −0.62; *p* = 0.008; GBM: r = −0.67; *p* = 0.002).

**Figure 5:**
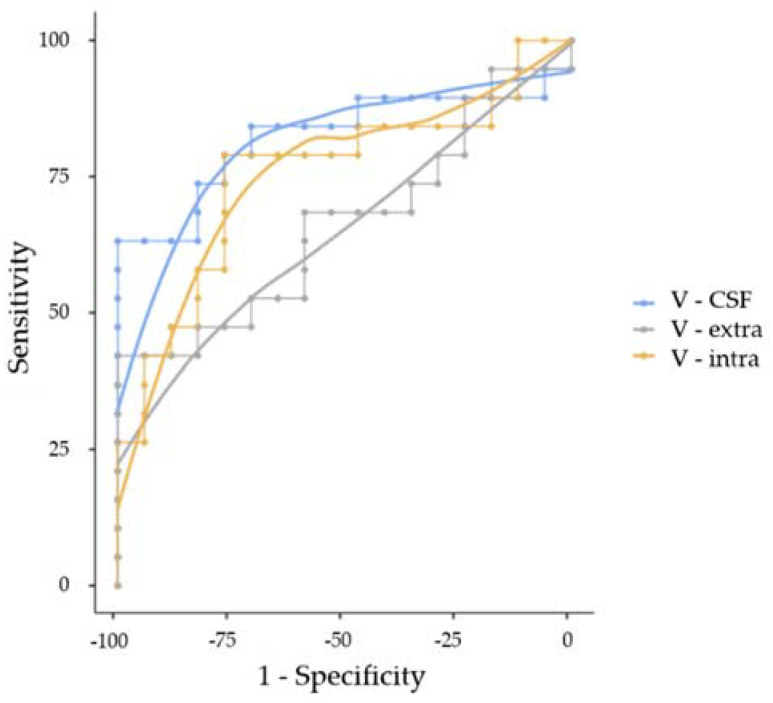
ROC curves of 19 patients with GBM and 17 with cerebral metastases showing a high predictive value of both perilesional T2 V-CSF (AUC 0.824) and V-intra (AUC 0.752) regarding the presence of a GBM.

Building on the systematic differences regarding free water content of perilesional T2 hyperintensities between metastases and GBM groups, we conducted a ROC analysis. A model equally weighted for sensitivity and specificity supported the affiliation to the GBM cohort for normalized V-CSF (sensitivity, 63.2%; specificity, 100.0%; PPV, 100.0%; NPV, 70.83%; AUC 0.824) when applying an estimated cutpoint of 3.509. V-intra appeared more sensitive but less specific (sensitivity, 78.95%; specificity, 76.47%; PPV, 78.95%; NPV, 76.47%; AUC 0.752), applying an optimal cutpoint of 0.224. The specificity of V-extra was high but sensitivity low (sensitivity, 42.11%; specificity, 100.0%; PPV, 100.0%; NPV, 60.71%; AUC 0.663) when applying a threshold of 0.989. ROC curves are presented in Figure 5.

### 3.4. Histopathology

**Figure 6:**
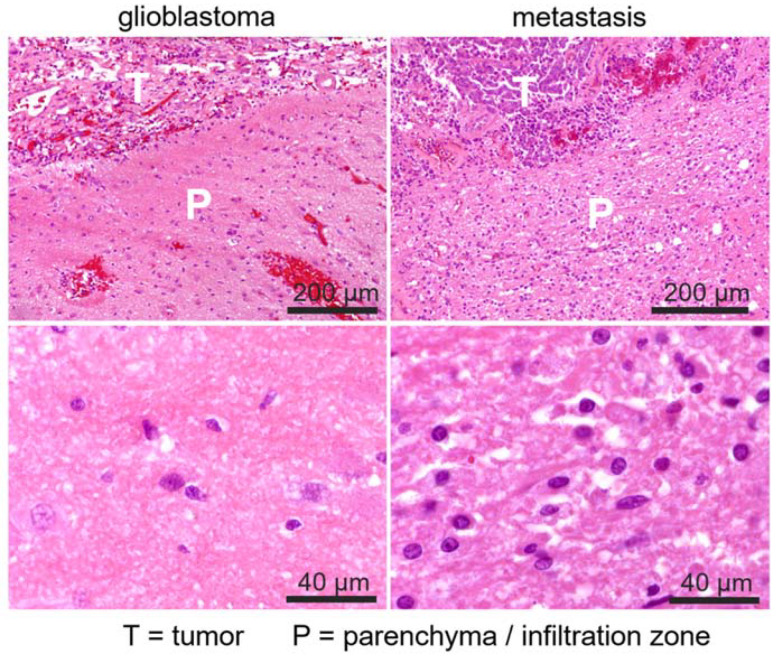
Example of histological sections in H&E staining in patients with IDHwt GBM (**left**) and metastasis of ovarian cancer (**right**). Images in the top row illustrate the immediate neighboring tumor (T) and adjacent parenchymal (P) areas in both samples. Microstructural loosening of the tissue in the surrounding white matter (P) appears prominently in the metastasis case, but is almost absent in glioblastoma, which is particularly visible in detailed images at higher magnification (400×, bottom row), and was interpreted as a sign of increased vasogenic edematization. Magnification bars indicate 200 µm (**top row**) and 40 µm (**bottom row**), respectively.

**Figure 7:**
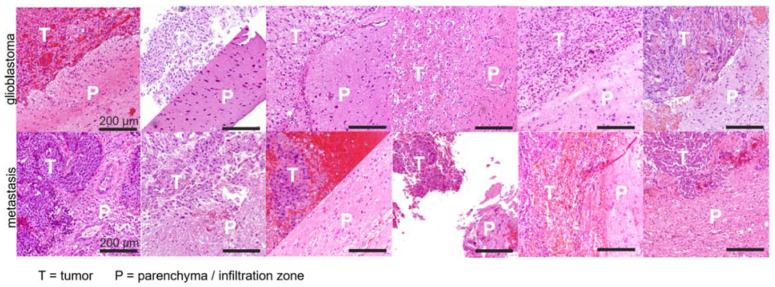
Exemplary histopathological sections in 6 patients with IDHwt GBM (**upper row**) and 6 patients with metastases (**lower row**). In each case, tumor area (T) and surrounding/infiltrating area (P) are obliquely juxtaposed. Metastases show an increased loosening of the surrounding immediate peritumoral tissue (see also Figure 6) in all cases by visual comparison with GBM. This image impression refers to the immediate peritumoral region. In some cases, the assessability was limited by the retrospective evaluation of surgery-related fragmented tissue. Magnification bars indicate 200 µm.

Histopathological assessment of H&E-stained biopsy material revealed signs of vasogenic edematization, including accentuated tissue loosening and increase in translucent areas in the perilesional parenchyma in all cases with metastases. By contrast, in GBM cases, signs of vasogenic edematization, if present, were unincisive or restricted to small areas of the assessed parenchyma. Exemplary images are shown in Figure 6 (detail histology) and Figure 7 (overview of assessed tumors).

## 4. Discussion

Noninvasive differentiation between GBM and brain metastases by conventional MRI remains difficult, as both entities show similar MRI phenotypes. Moreover, in GBM, stratification of non-Gd-enhancing tumor areas is of great clinical importance, as recurrences often occur in these parts of the brain. Assuming that perilesional T2 signal elevations in GBM and metastases differ histopathologically, we used a novel diffusion microstructure imaging approach and examined the obtained image parameters with respect to their diagnostic value.

Within perilesional T2 hyperintense areas of metastases, we measured a significant increase in the fraction of free water (V-CSF) compared to the GBM group. This effect persisted even after exclusion of patients pretreated with corticosteroids. Correspondingly, the volume fractions of the intra- and extra-axonal compartments (V-intra and V-extra) were significantly lower in the metastases group compared to the GBM group. Furthermore, a positive correlation between perilesional T2 volume and V-CSF in metastases was observed, which was not the case in GBM. For both brain metastases and GBM groups, a negative correlation between perilesional T2 volume and patient age was found. Concerning the presence of GBM, ROC analysis found high predictive values of both V-CSF (AUC 0.824) and V-intra (0.752) in the perilesional T2 area. The histopathological assessment revealed signs of vasogenic edematization in the perilesional parenchyma of all examined metastases cases, whereas this finding was unincisive or restricted to small areas in the GBM cases.

The finding of increased V-CSF in brain metastases fit well to the histopathologically based hypothesis that peritumoral edema around brain metastases is predominantly vasogenic [23], whereas GBM contains additional tumor infiltration [13,24]. While conventional DWI with ADC provides an estimate of the diffusion magnitude, diffusion tensor imaging (DTI) with its FA and MD parameters describes both the magnitude and directionality of diffusion [25]. The advanced diffusion techniques used so far, such as DTI, mostly focused on the FA parameter, which captures the degree of directionality of brain tracts and also correlates with cell density in gliomas. One study showed higher FA levels in the contrast-enhancing tumor fraction in glioblastomas than in brain metastases [26]. Furthermore, in a study that applied five different diffusion techniques to high-grade gliomas and metastases, increased FA values were found in the contrast-attenuated tumor portions in high-grade gliomas compared to metastases [16]. However, none of the applied diffusion techniques succeeded in measuring significant differences in perifocal T2 changes. This could be explained by the fact that the edematous changes would affect the measure of anisotropy and thus the differentiation based on FA and MD is compromised. The 3-compartment model of DMI could in theory allow quantification of distant cell infiltration, but this does not currently seem possible to us for several reasons: on the one hand, reliable differentiation of tumoral and non-tumoral glial cells is not possible, on the other hand, edematization (which also occurs in GBM) is accompanied by a redistribution of cellular compartments and probably also volume increase, which would have an additional impact on the distribution of cellular and noncellular compartments. Therefore, T2 volume was included in the analysis in our study, but it remains unclear to what extent volume effects in GBM influence the distribution of physiological and tumoral cellular structures or the indirect calculation of the parameters V-intra and V-extra. For this, further studies, ideally with quantitative immunohistopathological methods, would be useful.

DMI metrics were more widely distributed in the GBM group compared to the metastasis group. This could be explained by infiltrative GBM growth in non-contrast-enhancing areas and possibly higher intertumoral heterogeneity of GBM with varying extent of the vasogenic edema component, whereas in metastases the predominantly vasogenic edema could be more homogeneous at the cellular level.

The DMI technique has several potential advantages over NODDI. Violations of the NODDI constraints may induce confounding changes in actually independent parameters, which may lead to less specific results. In contrast, the applied Bayesian approach leads to much softer constraints and better interpretability in pathologically altered tissue. The main contribution to the dMRI signal at the cellular level is defined by microstructural indices such as the diffusion coefficients of the different cell compartments and their volume fractions. However, due to the coarse millimeter resolution of dMRI, much of the microscopic information is usually obscured by mesoscopic changes. The DMI technique disaggregates the microscopic cell properties from the effects of mesoscopic structures. The significantly faster examination time compared to NODDI allows the practical application even in clinical routine MRI examinations.

The negative correlation between patient age and T2 volume is difficult to interpret. The extent of neovascularization and vascular endothelial growth factor (VEGF) expression has been shown to influence the development of peritumoral edema in gliomas, meningiomas, and brain metastases [27,28]. Thus, increased VEGF expression is described to be associated with greater peritumoral edema [29], and increased VEGF expression was found more frequently in elderly GBM patients [30]. However, another study in patients with primary GBM did not find an association between age and edema size [31]. Additionally, a selection bias of patient age with the time of examination (which is indirectly related to the expression of the edema) cannot be excluded with absolute certainty. Thus, from our point of view, this finding remains not completely explicable.

This study has several limitations: First, the retrospective, monocentric design, the single 3T MRI and the in-house postprocessing pipeline lead to a relatively small number of patients included in this study. Nevertheless, the platform is freely available and further application, also in multicenter cohorts, is possible. Concerning the ROI-based measurements, mean values of both groups were compared and it remains an open question to what extent locoregional heterogeneity exists within the perilesional area. Moreover, diffusion metrics were normalized to whole-brain NAWM. On the one hand, tumor extension in GBM, even beyond the T2 hyperintense parenchyma has been reported in the past [32], on the other hand, we had to account for locoregional variability, which is relevant if normalization ROIs are placed for example within the corticospinal tract. In addition, some of the patients received corticosteroids before the MRI examination. Corticosteroids have been part of the standard treatment of peritumoral vasogenic edema in brain tumors for decades, as they reduce the permeability of tumor capillaries. A reduction in volume of both CE tumor and perilesional T2 changes have been reported for both GBM and metastasis cases [33]. However, we are not aware of exact prospective studies on the effect on quantitative imaging parameters of brain edema.

Approximately one-third of cases in both cohorts received corticosteroids and the observed effects did not differ after exclusion of these subgroups of patients. Whether the potentially different effects of corticosteroids on tumor tissue in the respective groups reflects on their properties in MRI, should ideally be investigated in prospective trials with larger patient groups and standardized corticosteroid doses and timing before imaging as the subgroups in our trial were too small to answer this question.

To our knowledge, our study is the first to use a novel diffusion microstructure imaging (DMI) sequence and was able to demonstrate significant differences in diffusion metrics between perifocal T2 areas in brain metastases and GBM. The methodology is promising in terms of microstructural characterization of brain tumors and has high diagnostic potential to pre-surgically differentiate GBM from metastases.

## 5. Conclusions

Diffusion microstructure imaging extends the capabilities of current advanced diffusion imaging techniques by allowing characterization of perilesional white matter T2 changes based on a 3-compartment model. In contrast to GBM, perilesional T2 changes surrounding metastases showed a significant increase in the free water fraction, which was also qualitatively reproduced in histopathological samples. This underscores the concept of a predominant vasogenic-related peritumoral edema around brain metastases, in contrast to perilesional edema and additional infiltrating tumor component in GBM. Thus, diffusion microstructure imaging may not only play an important role in distinguishing GBM from brain metastases but also to further characterize the microstructure of distant tumor components.

## Figures and Tables

**Table 1:** Patient characteristics and ROI (perilesional T2 hyperintense area)-derived diffusion metrics.

Heading	GBM	Metastasis	*p*-Value
*n*	19	17	
Sex (m/f)	10/9	9/8	
Age (years) (SD)	66.4 (14.1)	63.5 (11.8)	*p* = 0.509
Perifocal T2 volume (ml) (IQR)	20.7 (22.1)	19.5 (35.1)	*p* = 0.925
Presurgical steroid treatment	7/19 (36.8%)	6/17 (35.3%)	
V-intra (IQR)	0.25 (0.13)	0.20 (0.03)	*p* = 0.006
V-extra (IQR)	0.88 (0.30)	0.81 (0.18)	*p* = 0.024
V-CSF (IQR)	3.17 (1.26)	4.52 (0.86)	*p* < 0.001

Values are given in mean and SD or median and interquartile ranges (IQR).

## Data Availability

The anonymized data presented in this study are available on reasonable request from the corresponding author.

## Supplementary Materials

The following supporting information can be downloaded at: https://www.mdpi.com/article/10.3390/cancers14051155/s1, Table S1: MRI sequence parameters (3-Tesla MAGNETOM Prisma, Siemens Healthcare, Erlangen, Germany).
