# Supplementary material for: Diffusion Microstructure Imaging to Analyze Perilesional T2 Signal Changes in Brain Metastases and Glioblastomas
Source: Cancers (Basel). 2022 Feb 23;14(5):1155. doi: 10.3390/cancers14051155 (PMC8908999; doi:10.3390/cancers14051155)
Supplement: Supplementary file 1 [file cancers-14-01155-s001.zip › cancers-1557236-supplementary.pdf]

cancers

Supplementary Materials

Diffusion Microstructure Imaging to Analyze Perilesional T2 Signal Changes in Brain Metastases and Glioblastomas

Urs Würtemberger [1,*], Martin Diebold [2,3], Daniel Erny [2,4], Jonas A. Hosp [5], Oliver Schnell [6], Peter C. Reinacher [7,8], Alexander Rau [1,9], Elias Kellner [10], Marco Reisert [7,10], Horst Urbach [1] and Theo Demerath [1]

Department of Neuroradiology, Medical Center—University of Freiburg, Faculty of Medicine, University of Freiburg, 79106 Freiburg, Germany; alexander.rau@uniklinik-freiburg.de (A.R.); horst.urbach@uniklinik-freiburg.de (H.U.); theo.demerath@uniklinik-freiburg.de (T.D.)
[2] Institute of Neuropathology, Medical Center—University of Freiburg, Faculty of Medicine, University of Freiburg, 79106 Freiburg, Germany; martin.diebold@uniklinik-freiburg.de (M.D.); daniel.erny@uniklinik-freiburg.de (D.E.)
[3] IMM-PACT Clinician Scientist Program, Faculty of Medicine, University of Freiburg, 79106 Freiburg, Germany
[4] Berta-Ottenstein-Program for Advanced Clinician Scientists, Faculty of Medicine, University of Freiburg, 79106 Freiburg, Germany
Department of Neurology and Neurophysiology, Medical Center—University of Freiburg, Faculty of Medicine, University of Freiburg, 79106 Freiburg, Germany; jonas.hosp@uniklinik-freiburg.de
[6] Department of Neurosurgery, Medical Center—University of Freiburg, Faculty of Medicine, University of Freiburg, 79106 Freiburg, Germany; oliver.schnell@uniklinik-freiburg.de
[7] Department of Stereotactic and Functional Neurosurgery, Medical Center—University of Freiburg, Faculty of Medicine, University of Freiburg, 79106 Freiburg, Germany; peter.reinacher@uniklinik-freiburg.de (P.C.R.); marco.reisert@uniklinik-freiburg.de (M.R.)
[8] Fraunhofer Institute for Laser Technology, 52074 Aachen, Germany
[9] Department of Diagnostic and Interventional Radiology, Medical Center—University of Freiburg, Faculty of Medicine, University of Freiburg, 79106 Freiburg, Germany
[10] Department of Medical Physics, Medical Center—University of Freiburg, Faculty of Medicine, University of Freiburg, 79106 Freiburg, Germany; elias.kellner@uniklinik-freiburg.de
* Correspondence: urs.würtemberger@uniklinik-freiburg.de; Tel.: +49-761-270-51810; Fax: +49-761-270-51950

Table S1. MRI sequence parameters (3-Tesla MAGNETOM Prisma, Siemens Healthcare, Erlangen, Germany).

MRI Sequence	No. of Slices/ Thickness (mm)	Voxel Size (mm^3)	TI(ms)/TR(ms)/ TE(ms)/α(°)	Acquisition Time (Min:Sec)
**sag 3D FLAIR**	160/1	1 × 1 × 1	1800/5000/388/var	6:52
**sag 3D MPRAGE pre- and post-Gd**	192/1	1 × 1 × 1	1100/2500/2.82/7	3:58
**DTI/DMI**	42/3	1.5 × 1.5 × 3	-/2800/88.5/90	6:22

MPRAGE = Magnetization Prepared Rapid Gradient Echo, FLAIR = Fluid-Attenuated Inversion Recovery, DTI = Diffusion tensor Imaging, DMI= Diffusion Microstructure Imaging, TI = inversion time, TR = repetition time, TE = echo time, α = flip angle, var = variable flip angle.
